# (*S*,*R*,*Rp*)-*N*,*N*-Dimethyl-1-{2-[(1-phenyl­ethyl)amino­meth­yl]ferrocen­yl}ethanamine

**DOI:** 10.1107/S1600536809008861

**Published:** 2009-03-25

**Authors:** Xiu-Li Zheng, Jin-Ting Liu

**Affiliations:** a School of Chemistry and Chemical Engineering, Shandong University, Jinan 250100, People’s Republic of China

## Abstract

The title chiral ferrocene compound, [Fe(C_5_H_5_)(C_18_H_25_N_2_)], contains one planar and two central chiral centers. It is of inter­est with respect to asymmetric catalysis. The absolute configuration of the planar chirality is *Rp* at the ferrocene group and those of the two C chiral centers are *R* at the CH carbon of the ethanamine unit and *S* at the CH carbon of the phenyl­ethyl­amino substituent. In the ferrocenyl unit, the cyclo­penta­dienyl (Cp) rings are planar, with maximum deviations of 0.002 (2) Å for the substituted and 0.008 (3) Å for the unsubstituted Cp ring. The dihedral angle between the ring planes is 2.12 (15)° and the rings are twisted slightly from an eclipsed conformation by 7.06–7.60°.

## Related literature

For background to the chemistry of chiral ferrocene complexes, see: Togni (1996 ▶); Nishibayashi *et al.* (1996 ▶). For their use in asymmetric synthesis, see: Togni *et al.* (1994 ▶); Dai *et al.* (2003 ▶). For the potential of these compounds as ligands in asymmetric catalysis, see Nikolaides *et al.* (2008 ▶). For a related structure, see: Liu *et al.* (2007 ▶).
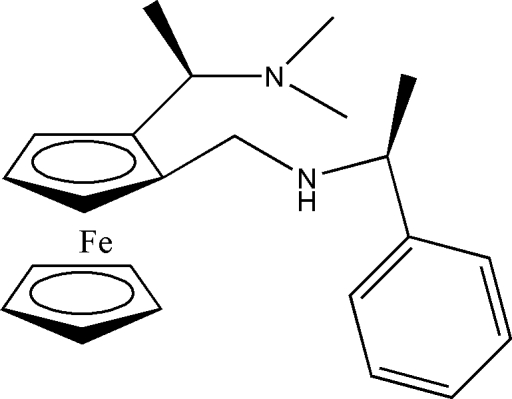


## Experimental

### 

#### Crystal data

[Fe(C_5_H_5_)(C_18_H_25_N_2_)]
*M* *_r_* = 390.34
Orthorhombic, 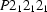

*a* = 7.2081 (1) Å
*b* = 16.5546 (3) Å
*c* = 17.6747 (2) Å
*V* = 2109.07 (5) Å^3^
*Z* = 4
Mo *K*α radiation
μ = 0.72 mm^−1^
*T* = 293 K
0.37 × 0.24 × 0.22 mm

#### Data collection

Bruker APEXII CCD area-detector diffractometer
Absorption correction: multi-scan (*SADABS*; Bruker, 2005 ▶) *T*_min_ = 0.776, *T*_max_ = 0.857
9974 measured reflections
4636 independent reflections
3817 reflections with *I* > 2σ(*I*)
*R*_int_ = 0.022

#### Refinement

*R*[*F* ^2^ > 2σ(*F* ^2^)] = 0.032
*wR*(*F* ^2^) = 0.085
*S* = 1.04
4636 reflections
240 parameters
H-atom parameters constrained
Δρ_max_ = 0.31 e Å^−3^
Δρ_min_ = −0.32 e Å^−3^
Absolute structure: Flack (1983 ▶), 1859 Friedel pairs
Flack parameter: 0.010 (17)

### 

Data collection: *APEX2* (Bruker, 2005 ▶); cell refinement: *APEX2* and *SAINT* (Bruker, 2005 ▶); data reduction: *SAINT*; program(s) used to solve structure: *SIR97* (Altomare *et al.*, 1999 ▶); program(s) used to refine structure: *SHELXL97* (Sheldrick, 2008 ▶); molecular graphics: *SHELXTL* (Sheldrick, 2008 ▶); software used to prepare material for publication: *WinGX* (Farrugia, 1999 ▶).

## Supplementary Material

Crystal structure: contains datablocks I, global. DOI: 10.1107/S1600536809008861/sj2592sup1.cif

Structure factors: contains datablocks I. DOI: 10.1107/S1600536809008861/sj2592Isup2.hkl

Additional supplementary materials:  crystallographic information; 3D view; checkCIF report

## Figures and Tables

**Table 1:** Selected torsion angles (°)

C1—*Cg*1—*Cg*2—C6	7.60	C4—*Cg*1—*Cg*2—C9	7.51
C2—*Cg*1—*Cg*2—C7	7.06	C5—*Cg*1—*Cg*2—C10	7.16
C3—*Cg*1—*Cg*2—C8	7.38		

*Cg*1 is the centroid of the C1–C5 Cp ring and *Cg*2 is the centroid of the C6–C10 Cp ring.

**Fig. 1.**
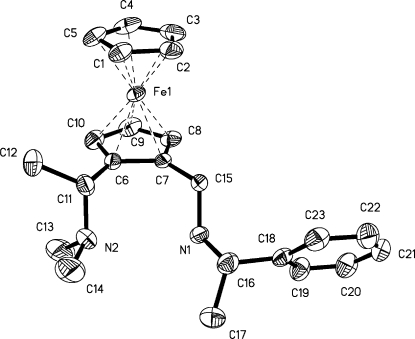
The molecular structure of the title compound, with displacement ellipsoids drawn at the 50% probabiltiy level and H atoms omitted.

## supplementary crystallographic information

### Comment

Ferrocenes with chirality are increasing their importance as chiral ligands in asymmetric catalysis (Togni, 1996, Nishibayashi *et al.*, 1996). Especially ferrocene-based ligands incorporating both planar and central chirality are very important and some of them have already been applied in hydrogenation, allylic alkylation, and hydroboration (Togni *et al.*, 1994, Dai *et al.*, 2003). We are interested in the synthesis and the fascinating properties of chiral ferrocenes with both central and planar chirality (Liu *et al.*, 2007).

The molecular structure of the title compound is shown in Fig. 1. The absolute configuration of the molecule is *Rp* at the ferrocene group, *R *and *S *at the asymmetric carbon atoms C11 and C16 respectively. Both N atoms have a pyramidal geometry with C—N—C angles between 112.0 (2) and 113.95 (18)°. The N lone-pairs are not involved in short intra- or intermolecular interactions. The two N atoms lie on the same coordination plane and may be available to coordinate to metal ions as bidentate ligands or to act as a ligand for asymmetric catalysis (Nikolaides *et al.*, 2008).

In the molecule, the cyclopentadienyl(Cp) rings are almost parallel with a dihedral angle of 2.12 (15)° between the Cp ring planes. The Cp rings are twisted slightly from the eclipsed conformation. The values of torsion angles of the two Cp rings (C—*Cg*1—*Cg*2—C) are in the range 7.06–7.61° (Table 1). The benzene and Cp rings are obviously not parallel. The dihedral angle between the substituted Cp ring and the benzene ring is 53.59 (13)°. The Fe—C bond distances within the ferrocene group are in the range of 2.023 (3)–2.046 (2)Å for the unsubstituted cyclopentadienyl (Cp) ring (C1–C5) and 2.024 (2)–2.052 (2)Å for the substituted Cp ring (C6–C10).

### Experimental

(*R,Sp*)-2-[1-(dimethylamino)ethyl]ferrocenylaldehyde(0.285 g, 1 mmol) was dissolved in anhydrous chloroform (30 ml) and (*S*)-1-phenylethanamine(0.121 mg, 1 mmol) was added. The red solution was refluxed under a nitrogen atmosphere for 2 h. After removing the solvent, the residue was dissolved in 30 ml dry methanol. 0.160 g NaBH_4_ were added in small portions. The mixture was stirred for 30 min, then 20 ml, 1 mol/*L* aqueous NaOH was added and the mixture was extracted with CHCl_3_(3×30 ml). The combined organic phase was dried with anhydrous NaCO_3_. The solvent was evaporated *in vacuo* and the residue was purified by column chromatography (silica gel; hexane-ethyl acetate, 6:2) giving the title compound as yellow crystals (0.356 mg, 91.3%). Single crystals were grown from hexane- ethylacetate (2:1) solution at room temperature.

### Refinement

All H atoms were placed in geometrically calculated positions and refined using a riding model with C—H = 0.98 Å (for the C_5_H_5_ groups), 0.93 Å (for the C_6_H_6_groups) and 0.96 Å (for CH_3_ groups), their isotropic displacement parameters were set to 1.2 times (1.5times for CH_3_ groups) the equivalent displacement parameters of their parent atoms. The absolute configuration of the structure was determined from the diffraction data using 1859 Friedel pairs with the Flack parameter (Flack, 1983) 0.010 (17).

### Crystal data

**Table:**

[Fe(C_5_H_5_)(C_18_H_25_N_2_)]	*D*_x_ = 1.229 Mg m^−^^3^
*M**_r_* = 390.34	Melting point: 355 K
Orthorhombic, *P*2_1_2_1_2_1_	Mo *K*α radiation, λ = 0.71073 Å
Hall symbol: P 2ac 2ab	Cell parameters from 4056 reflections
*a* = 7.2081 (1) Å	θ = 2.5–26.6°
*b* = 16.5546 (3) Å	µ = 0.72 mm^−^^1^
*c* = 17.6747 (2) Å	*T* = 293 K
*V* = 2109.07 (5) Å^3^	Prism, orange
*Z* = 4	0.37 × 0.24 × 0.22 mm
*F*(000) = 832	

### Data collection

**Table:**

Bruker APEXII CCD area-detector diffractometer	4636 independent reflections
Radiation source: fine-focus sealed tube	3817 reflections with *I* > 2σ(*I*)
graphite	*R*_int_ = 0.022
φ and ω scans	θ_max_ = 27.5°, θ_min_ = 1.7°
Absorption correction: multi-scan (*SADABS*; Bruker, 2005)	*h* = −9→9
*T*_min_ = 0.776, *T*_max_ = 0.857	*k* = −17→21
9974 measured reflections	*l* = −22→19

### Refinement

**Table:**

Refinement on *F*^2^	Hydrogen site location: inferred from neighbouring sites
Least-squares matrix: full	H-atom parameters constrained
*R*[*F*^2^ > 2σ(*F*^2^)] = 0.032	*w* = 1/[σ^2^(*F*_o_^2^) + (0.0459*P*)^2^] where *P* = (*F*_o_^2^ + 2*F*_c_^2^)/3
*wR*(*F*^2^) = 0.085	(Δ/σ)_max_ = 0.001
*S* = 1.04	Δρ_max_ = 0.31 e Å^−^^3^
4636 reflections	Δρ_min_ = −0.32 e Å^−^^3^
240 parameters	Extinction correction: *SHELXL97* (Sheldrick, 2008), Fc^*^=kFc[1+0.001xFc^2^λ^3^/sin(2θ)]^-1/4^
0 restraints	Extinction coefficient: 0.0097 (9)
Primary atom site location: structure-invariant direct methods	Absolute structure: Flack (1983), 1859 Friedel pairs
Secondary atom site location: difference Fourier map	Flack parameter: 0.010 (17)

### Special details

**Table:**

Geometry. All e.s.d.'s (except the e.s.d. in the dihedral angle between two l.s. planes) are estimated using the full covariance matrix. The cell e.s.d.'s are taken into account individually in the estimation of e.s.d.'s in distances, angles and torsion angles; correlations between e.s.d.'s in cell parameters are only used when they are defined by crystal symmetry. An approximate (isotropic) treatment of cell e.s.d.'s is used for estimating e.s.d.'s involving l.s. planes.
Refinement. Refinement of *F*^2^ against ALL reflections. The weighted *R*-factor *wR* and goodness of fit *S* are based on *F*^2^, conventional *R*-factors *R* are based on *F*, with *F* set to zero for negative *F*^2^. The threshold expression of *F*^2^ > σ(*F*^2^) is used only for calculating *R*-factors(gt) *etc*. and is not relevant to the choice of reflections for refinement. *R*-factors based on *F*^2^ are statistically about twice as large as those based on *F*, and *R*- factors based on ALL data will be even larger.

### Fractional atomic coordinates and isotropic or equivalent isotropic displacement parameters (Å^2^)

**Table:**

	*x*	*y*	*z*	*U*_iso_*/*U*_eq_	
C1	1.0099 (3)	0.12997 (16)	0.17507 (15)	0.0575 (6)	
H1	1.0734	0.1596	0.2155	0.069*	
C2	0.8903 (4)	0.06312 (16)	0.18440 (17)	0.0640 (7)	
H2	0.8560	0.0375	0.2324	0.077*	
C3	0.8321 (4)	0.03934 (17)	0.1114 (2)	0.0773 (8)	
H3	0.7485	−0.0057	0.1000	0.093*	
C4	0.9105 (4)	0.0916 (2)	0.05836 (17)	0.0750 (8)	
H4	0.8926	0.0890	0.0035	0.090*	
C5	1.0207 (3)	0.14668 (19)	0.09645 (15)	0.0633 (7)	
H5	1.0932	0.1901	0.0731	0.076*	
C6	0.6597 (3)	0.25943 (12)	0.18108 (11)	0.0425 (5)	
C7	0.5401 (3)	0.19310 (12)	0.20037 (11)	0.0392 (4)	
C8	0.4708 (3)	0.16030 (15)	0.13168 (14)	0.0525 (5)	
H8	0.3871	0.1139	0.1273	0.063*	
C9	0.5447 (3)	0.20488 (18)	0.07092 (13)	0.0609 (7)	
H9	0.5200	0.1952	0.0172	0.073*	
C10	0.6594 (3)	0.26565 (15)	0.10043 (13)	0.0564 (6)	
H10	0.7289	0.3054	0.0707	0.068*	
C11	0.7553 (4)	0.31317 (12)	0.23737 (12)	0.0550 (5)	
H11	0.7953	0.2780	0.2789	0.066*	
C12	0.9319 (4)	0.35294 (18)	0.2063 (2)	0.0882 (10)	
H12A	0.9009	0.3859	0.1634	0.132*	
H12B	1.0182	0.3118	0.1911	0.132*	
H12C	0.9870	0.3860	0.2448	0.132*	
C13	0.5480 (6)	0.4271 (2)	0.2146 (3)	0.1220 (15)	
H13A	0.4445	0.4559	0.2354	0.183*	
H13B	0.5093	0.3987	0.1699	0.183*	
H13C	0.6447	0.4645	0.2017	0.183*	
C14	0.6865 (6)	0.4093 (2)	0.3382 (2)	0.1248 (16)	
H14A	0.7824	0.4469	0.3246	0.187*	
H14B	0.7360	0.3695	0.3721	0.187*	
H14C	0.5868	0.4377	0.3625	0.187*	
C15	0.4913 (3)	0.16768 (12)	0.27843 (12)	0.0461 (5)	
H15A	0.4376	0.1140	0.2772	0.055*	
H15B	0.6029	0.1655	0.3091	0.055*	
C16	0.3378 (4)	0.21395 (16)	0.39411 (14)	0.0633 (7)	
H16	0.4616	0.2072	0.4161	0.076*	
C17	0.2528 (6)	0.28883 (17)	0.42764 (16)	0.1024 (11)	
H17A	0.3336	0.3340	0.4191	0.154*	
H17B	0.2356	0.2813	0.4810	0.154*	
H17C	0.1349	0.2989	0.4042	0.154*	
C18	0.2250 (3)	0.13936 (13)	0.41281 (11)	0.0509 (5)	
C19	0.0457 (4)	0.12998 (17)	0.38743 (14)	0.0650 (7)	
H19	−0.0058	0.1695	0.3565	0.078*	
C20	−0.0590 (4)	0.06375 (19)	0.40671 (17)	0.0758 (8)	
H20	−0.1794	0.0589	0.3883	0.091*	
C21	0.0113 (5)	0.00555 (17)	0.45218 (17)	0.0759 (8)	
H21	−0.0612	−0.0384	0.4663	0.091*	
C22	0.1906 (5)	0.01226 (19)	0.47722 (16)	0.0807 (10)	
H22	0.2409	−0.0280	0.5077	0.097*	
C23	0.2971 (4)	0.07837 (17)	0.45754 (14)	0.0668 (7)	
H23	0.4189	0.0819	0.4746	0.080*	
Fe1	0.75311 (4)	0.155327 (15)	0.131986 (14)	0.04165 (10)	
N1	0.3592 (3)	0.22386 (11)	0.31260 (10)	0.0502 (5)	
H1A	0.2996	0.2595	0.2869	0.060*	
N2	0.6172 (4)	0.36957 (12)	0.27003 (14)	0.0728 (6)	

### Atomic displacement parameters (Å^2^)

**Table:**

	*U*^11^	*U*^22^	*U*^33^	*U*^12^	*U*^13^	*U*^23^
C1	0.0306 (11)	0.0782 (18)	0.0636 (15)	0.0089 (11)	−0.0034 (10)	−0.0003 (12)
C2	0.0506 (14)	0.0629 (15)	0.0785 (17)	0.0228 (12)	0.0044 (12)	0.0085 (13)
C3	0.0554 (15)	0.0600 (16)	0.116 (2)	0.0148 (12)	−0.0005 (16)	−0.0323 (16)
C4	0.0477 (15)	0.109 (2)	0.0685 (17)	0.0210 (15)	0.0076 (13)	−0.0260 (17)
C5	0.0323 (11)	0.0912 (19)	0.0665 (15)	0.0067 (12)	0.0094 (11)	−0.0015 (15)
C6	0.0398 (11)	0.0395 (11)	0.0483 (11)	0.0034 (9)	0.0055 (9)	0.0042 (9)
C7	0.0287 (10)	0.0402 (10)	0.0485 (11)	0.0030 (8)	0.0048 (8)	−0.0034 (9)
C8	0.0258 (9)	0.0647 (13)	0.0668 (14)	0.0042 (9)	−0.0048 (10)	−0.0160 (15)
C9	0.0439 (13)	0.0933 (19)	0.0457 (13)	0.0131 (13)	−0.0078 (11)	−0.0003 (13)
C10	0.0504 (13)	0.0651 (15)	0.0538 (13)	0.0086 (11)	0.0042 (12)	0.0160 (12)
C11	0.0559 (12)	0.0421 (10)	0.0669 (13)	−0.0068 (12)	−0.0005 (15)	−0.0005 (9)
C12	0.070 (2)	0.0742 (19)	0.120 (3)	−0.0304 (15)	0.0168 (18)	−0.0116 (19)
C13	0.143 (4)	0.065 (2)	0.157 (4)	0.043 (2)	0.039 (3)	0.013 (2)
C14	0.122 (3)	0.115 (3)	0.137 (3)	−0.041 (2)	0.029 (3)	−0.079 (2)
C15	0.0461 (12)	0.0376 (11)	0.0545 (12)	0.0032 (9)	0.0117 (10)	0.0045 (9)
C16	0.0703 (16)	0.0692 (16)	0.0503 (13)	−0.0099 (13)	0.0105 (12)	−0.0073 (12)
C17	0.148 (3)	0.0723 (18)	0.0865 (19)	−0.013 (3)	0.045 (3)	−0.0284 (15)
C18	0.0549 (15)	0.0620 (13)	0.0360 (9)	0.0027 (11)	0.0093 (10)	−0.0014 (8)
C19	0.0621 (16)	0.0756 (17)	0.0574 (15)	0.0046 (13)	0.0012 (12)	0.0194 (12)
C20	0.0616 (17)	0.092 (2)	0.0741 (18)	−0.0082 (15)	0.0021 (14)	0.0083 (16)
C21	0.090 (2)	0.0652 (17)	0.0727 (17)	−0.0054 (15)	0.0308 (17)	0.0090 (14)
C22	0.098 (3)	0.0750 (18)	0.0695 (17)	0.0160 (16)	0.0104 (15)	0.0252 (14)
C23	0.0604 (18)	0.0846 (18)	0.0555 (13)	0.0105 (13)	0.0007 (12)	0.0056 (13)
Fe1	0.02711 (14)	0.05359 (17)	0.04423 (15)	0.00363 (15)	0.00133 (16)	−0.00439 (12)
N1	0.0546 (11)	0.0482 (10)	0.0477 (10)	0.0096 (8)	0.0138 (9)	0.0055 (8)
N2	0.0835 (16)	0.0471 (12)	0.0877 (15)	−0.0094 (11)	0.0198 (14)	−0.0162 (11)

### Geometric parameters (Å, °)

**Table:**

C1—C2	1.413 (4)	C12—H12A	0.9600
C1—C5	1.419 (4)	C12—H12B	0.9600
C1—Fe1	2.045 (2)	C12—H12C	0.9600
C1—H1	0.9800	C13—N2	1.454 (4)
C2—C3	1.412 (4)	C13—H13A	0.9600
C2—Fe1	2.041 (2)	C13—H13B	0.9600
C2—H2	0.9800	C13—H13C	0.9600
C3—C4	1.395 (4)	C14—N2	1.460 (4)
C3—Fe1	2.036 (3)	C14—H14A	0.9600
C3—H3	0.9800	C14—H14B	0.9600
C4—C5	1.384 (4)	C14—H14C	0.9600
C4—Fe1	2.024 (3)	C15—N1	1.462 (2)
C4—H4	0.9800	C15—H15A	0.9700
C5—Fe1	2.033 (2)	C15—H15B	0.9700
C5—H5	0.9800	C16—N1	1.458 (3)
C6—C10	1.429 (3)	C16—C17	1.504 (4)
C6—C7	1.437 (3)	C16—C18	1.515 (3)
C6—C11	1.502 (3)	C16—H16	0.9800
C6—Fe1	2.043 (2)	C17—H17A	0.9600
C7—C8	1.421 (3)	C17—H17B	0.9600
C7—C15	1.485 (3)	C17—H17C	0.9600
C7—Fe1	2.0514 (18)	C18—C19	1.377 (3)
C8—C9	1.408 (4)	C18—C23	1.384 (3)
C8—Fe1	2.0368 (19)	C19—C20	1.374 (4)
C8—H8	0.9800	C19—H19	0.9300
C9—C10	1.403 (4)	C20—C21	1.353 (4)
C9—Fe1	2.024 (2)	C20—H20	0.9300
C9—H9	0.9800	C21—C22	1.371 (4)
C10—Fe1	2.025 (2)	C21—H21	0.9300
C10—H10	0.9800	C22—C23	1.381 (4)
C11—N2	1.482 (3)	C22—H22	0.9300
C11—C12	1.535 (3)	C23—H23	0.9300
C11—H11	0.9800	N1—H1A	0.8600
			
C2—C1—C5	107.5 (2)	C7—C15—H15A	109.4
C2—C1—Fe1	69.61 (14)	N1—C15—H15B	109.4
C5—C1—Fe1	69.18 (14)	C7—C15—H15B	109.4
C2—C1—H1	126.3	H15A—C15—H15B	108.0
C5—C1—H1	126.3	N1—C16—C17	109.9 (2)
Fe1—C1—H1	126.3	N1—C16—C18	111.36 (19)
C3—C2—C1	107.0 (3)	C17—C16—C18	111.5 (2)
C3—C2—Fe1	69.52 (16)	N1—C16—H16	108.0
C1—C2—Fe1	69.94 (14)	C17—C16—H16	108.0
C3—C2—H2	126.5	C18—C16—H16	108.0
C1—C2—H2	126.5	C16—C17—H17A	109.5
Fe1—C2—H2	126.5	C16—C17—H17B	109.5
C4—C3—C2	108.7 (3)	H17A—C17—H17B	109.5
C4—C3—Fe1	69.44 (17)	C16—C17—H17C	109.5
C2—C3—Fe1	69.94 (14)	H17A—C17—H17C	109.5
C4—C3—H3	125.6	H17B—C17—H17C	109.5
C2—C3—H3	125.6	C19—C18—C23	117.2 (2)
Fe1—C3—H3	125.6	C19—C18—C16	121.6 (2)
C5—C4—C3	108.3 (3)	C23—C18—C16	121.2 (2)
C5—C4—Fe1	70.42 (15)	C20—C19—C18	121.7 (3)
C3—C4—Fe1	70.36 (15)	C20—C19—H19	119.2
C5—C4—H4	125.8	C18—C19—H19	119.2
C3—C4—H4	125.8	C21—C20—C19	120.6 (3)
Fe1—C4—H4	125.8	C21—C20—H20	119.7
C4—C5—C1	108.5 (3)	C19—C20—H20	119.7
C4—C5—Fe1	69.67 (14)	C20—C21—C22	119.2 (3)
C1—C5—Fe1	70.10 (13)	C20—C21—H21	120.4
C4—C5—H5	125.8	C22—C21—H21	120.4
C1—C5—H5	125.8	C21—C22—C23	120.5 (3)
Fe1—C5—H5	125.8	C21—C22—H22	119.8
C10—C6—C7	106.91 (19)	C23—C22—H22	119.8
C10—C6—C11	128.2 (2)	C22—C23—C18	120.9 (3)
C7—C6—C11	124.79 (18)	C22—C23—H23	119.6
C10—C6—Fe1	68.76 (13)	C18—C23—H23	119.6
C7—C6—Fe1	69.75 (11)	C4—Fe1—C9	106.55 (12)
C11—C6—Fe1	129.02 (16)	C4—Fe1—C10	118.70 (12)
C8—C7—C6	107.48 (18)	C9—Fe1—C10	40.54 (10)
C8—C7—C15	127.04 (19)	C4—Fe1—C5	39.90 (11)
C6—C7—C15	125.40 (18)	C9—Fe1—C5	124.61 (11)
C8—C7—Fe1	69.11 (11)	C10—Fe1—C5	107.13 (11)
C6—C7—Fe1	69.16 (11)	C4—Fe1—C3	40.20 (12)
C15—C7—Fe1	129.67 (14)	C9—Fe1—C3	119.66 (12)
C9—C8—C7	108.6 (2)	C10—Fe1—C3	153.46 (13)
C9—C8—Fe1	69.22 (13)	C5—Fe1—C3	67.24 (13)
C7—C8—Fe1	70.22 (11)	C4—Fe1—C8	125.42 (11)
C9—C8—H8	125.7	C9—Fe1—C8	40.57 (10)
C7—C8—H8	125.7	C10—Fe1—C8	68.27 (10)
Fe1—C8—H8	125.7	C5—Fe1—C8	161.76 (11)
C10—C9—C8	108.4 (2)	C3—Fe1—C8	108.50 (11)
C10—C9—Fe1	69.80 (13)	C4—Fe1—C2	68.29 (12)
C8—C9—Fe1	70.22 (13)	C9—Fe1—C2	154.81 (11)
C10—C9—H9	125.8	C10—Fe1—C2	163.91 (11)
C8—C9—H9	125.8	C5—Fe1—C2	68.16 (12)
Fe1—C9—H9	125.8	C3—Fe1—C2	40.54 (12)
C9—C10—C6	108.7 (2)	C8—Fe1—C2	121.03 (11)
C9—C10—Fe1	69.66 (14)	C4—Fe1—C6	153.86 (12)
C6—C10—Fe1	70.11 (13)	C9—Fe1—C6	68.90 (9)
C9—C10—H10	125.7	C10—Fe1—C6	41.12 (9)
C6—C10—H10	125.7	C5—Fe1—C6	120.20 (11)
Fe1—C10—H10	125.7	C3—Fe1—C6	164.47 (11)
N2—C11—C6	108.8 (2)	C8—Fe1—C6	68.77 (9)
N2—C11—C12	115.25 (19)	C2—Fe1—C6	126.70 (10)
C6—C11—C12	113.4 (2)	C4—Fe1—C1	67.97 (11)
N2—C11—H11	106.2	C9—Fe1—C1	162.51 (11)
C6—C11—H11	106.2	C10—Fe1—C1	126.11 (11)
C12—C11—H11	106.2	C5—Fe1—C1	40.72 (10)
C11—C12—H12A	109.5	C3—Fe1—C1	67.64 (11)
C11—C12—H12B	109.5	C8—Fe1—C1	156.02 (11)
H12A—C12—H12B	109.5	C2—Fe1—C1	40.45 (11)
C11—C12—H12C	109.5	C6—Fe1—C1	108.25 (10)
H12A—C12—H12C	109.5	C4—Fe1—C7	163.22 (11)
H12B—C12—H12C	109.5	C9—Fe1—C7	68.60 (9)
N2—C13—H13A	109.5	C10—Fe1—C7	68.78 (9)
N2—C13—H13B	109.5	C5—Fe1—C7	155.95 (10)
H13A—C13—H13B	109.5	C3—Fe1—C7	127.02 (11)
N2—C13—H13C	109.5	C8—Fe1—C7	40.67 (9)
H13A—C13—H13C	109.5	C2—Fe1—C7	108.85 (10)
H13B—C13—H13C	109.5	C6—Fe1—C7	41.09 (8)
N2—C14—H14A	109.5	C1—Fe1—C7	121.37 (9)
N2—C14—H14B	109.5	C16—N1—C15	113.94 (19)
H14A—C14—H14B	109.5	C16—N1—H1A	123.0
N2—C14—H14C	109.5	C15—N1—H1A	123.0
H14A—C14—H14C	109.5	C13—N2—C14	112.2 (3)
H14B—C14—H14C	109.5	C13—N2—C11	112.3 (2)
N1—C15—C7	110.97 (16)	C14—N2—C11	112.0 (3)
N1—C15—H15A	109.4		
			
C5—C1—C2—C3	0.9 (3)	C1—C5—Fe1—C7	49.8 (3)
Fe1—C1—C2—C3	59.90 (17)	C2—C3—Fe1—C4	120.1 (2)
C5—C1—C2—Fe1	−59.04 (17)	C4—C3—Fe1—C9	80.4 (2)
C1—C2—C3—C4	−1.4 (3)	C2—C3—Fe1—C9	−159.44 (15)
Fe1—C2—C3—C4	58.77 (19)	C4—C3—Fe1—C10	45.2 (3)
C1—C2—C3—Fe1	−60.17 (17)	C2—C3—Fe1—C10	165.3 (2)
C2—C3—C4—C5	1.4 (3)	C4—C3—Fe1—C5	−37.59 (16)
Fe1—C3—C4—C5	60.48 (19)	C2—C3—Fe1—C5	82.53 (18)
C2—C3—C4—Fe1	−59.08 (18)	C4—C3—Fe1—C8	123.44 (17)
C3—C4—C5—C1	−0.9 (3)	C2—C3—Fe1—C8	−116.43 (17)
Fe1—C4—C5—C1	59.58 (18)	C4—C3—Fe1—C2	−120.1 (2)
C3—C4—C5—Fe1	−60.44 (19)	C4—C3—Fe1—C6	−159.4 (3)
C2—C1—C5—C4	0.0 (3)	C2—C3—Fe1—C6	−39.3 (5)
Fe1—C1—C5—C4	−59.32 (18)	C4—C3—Fe1—C1	−81.85 (18)
C2—C1—C5—Fe1	59.31 (17)	C2—C3—Fe1—C1	38.27 (16)
C10—C6—C7—C8	0.3 (2)	C4—C3—Fe1—C7	164.90 (15)
C11—C6—C7—C8	177.2 (2)	C2—C3—Fe1—C7	−74.98 (19)
Fe1—C6—C7—C8	−58.68 (13)	C9—C8—Fe1—C4	−72.9 (2)
C10—C6—C7—C15	−176.47 (19)	C7—C8—Fe1—C4	167.16 (16)
C11—C6—C7—C15	0.5 (3)	C7—C8—Fe1—C9	−119.9 (2)
Fe1—C6—C7—C15	124.58 (19)	C9—C8—Fe1—C10	37.60 (15)
C10—C6—C7—Fe1	58.96 (15)	C7—C8—Fe1—C10	−82.29 (14)
C11—C6—C7—Fe1	−124.1 (2)	C9—C8—Fe1—C5	−41.1 (4)
C6—C7—C8—C9	−0.1 (2)	C7—C8—Fe1—C5	−161.0 (3)
C15—C7—C8—C9	176.6 (2)	C9—C8—Fe1—C3	−114.31 (18)
Fe1—C7—C8—C9	−58.76 (15)	C7—C8—Fe1—C3	125.79 (15)
C6—C7—C8—Fe1	58.71 (14)	C9—C8—Fe1—C2	−157.09 (16)
C15—C7—C8—Fe1	−124.6 (2)	C7—C8—Fe1—C2	83.01 (16)
C7—C8—C9—C10	−0.2 (3)	C9—C8—Fe1—C6	81.95 (16)
Fe1—C8—C9—C10	−59.58 (17)	C7—C8—Fe1—C6	−37.95 (12)
C7—C8—C9—Fe1	59.38 (15)	C9—C8—Fe1—C1	169.3 (2)
C8—C9—C10—C6	0.4 (3)	C7—C8—Fe1—C1	49.4 (3)
Fe1—C9—C10—C6	−59.47 (16)	C9—C8—Fe1—C7	119.9 (2)
C8—C9—C10—Fe1	59.84 (17)	C3—C2—Fe1—C4	−36.93 (17)
C7—C6—C10—C9	−0.4 (3)	C1—C2—Fe1—C4	81.06 (18)
C11—C6—C10—C9	−177.2 (2)	C3—C2—Fe1—C9	45.8 (3)
Fe1—C6—C10—C9	59.19 (17)	C1—C2—Fe1—C9	163.8 (2)
C7—C6—C10—Fe1	−59.59 (14)	C3—C2—Fe1—C10	−155.9 (3)
C11—C6—C10—Fe1	123.6 (2)	C1—C2—Fe1—C10	−37.9 (4)
C10—C6—C11—N2	101.0 (3)	C3—C2—Fe1—C5	−80.05 (19)
C7—C6—C11—N2	−75.2 (3)	C1—C2—Fe1—C5	37.95 (16)
Fe1—C6—C11—N2	−166.40 (16)	C1—C2—Fe1—C3	118.0 (2)
C10—C6—C11—C12	−28.7 (3)	C3—C2—Fe1—C8	82.3 (2)
C7—C6—C11—C12	155.1 (2)	C1—C2—Fe1—C8	−159.69 (15)
Fe1—C6—C11—C12	63.9 (3)	C3—C2—Fe1—C6	167.79 (17)
C8—C7—C15—N1	−101.7 (2)	C1—C2—Fe1—C6	−74.21 (19)
C6—C7—C15—N1	74.4 (3)	C3—C2—Fe1—C1	−118.0 (2)
Fe1—C7—C15—N1	165.68 (15)	C3—C2—Fe1—C7	125.43 (17)
N1—C16—C18—C19	−59.7 (3)	C1—C2—Fe1—C7	−116.57 (16)
C17—C16—C18—C19	63.5 (3)	C10—C6—Fe1—C4	47.4 (3)
N1—C16—C18—C23	121.7 (2)	C7—C6—Fe1—C4	165.9 (2)
C17—C16—C18—C23	−115.2 (3)	C11—C6—Fe1—C4	−75.2 (3)
C23—C18—C19—C20	1.2 (4)	C10—C6—Fe1—C9	−37.20 (15)
C16—C18—C19—C20	−177.5 (2)	C7—C6—Fe1—C9	81.22 (13)
C18—C19—C20—C21	0.6 (4)	C11—C6—Fe1—C9	−159.8 (2)
C19—C20—C21—C22	−1.8 (5)	C7—C6—Fe1—C10	118.42 (19)
C20—C21—C22—C23	1.3 (4)	C11—C6—Fe1—C10	−122.6 (2)
C21—C22—C23—C18	0.5 (4)	C10—C6—Fe1—C5	81.47 (17)
C19—C18—C23—C22	−1.7 (4)	C7—C6—Fe1—C5	−160.10 (13)
C16—C18—C23—C22	177.0 (2)	C11—C6—Fe1—C5	−41.2 (2)
C5—C4—Fe1—C9	124.64 (19)	C10—C6—Fe1—C3	−163.6 (3)
C3—C4—Fe1—C9	−116.63 (18)	C7—C6—Fe1—C3	−45.1 (4)
C5—C4—Fe1—C10	82.5 (2)	C11—C6—Fe1—C3	73.8 (4)
C3—C4—Fe1—C10	−158.81 (17)	C10—C6—Fe1—C8	−80.85 (16)
C3—C4—Fe1—C5	118.7 (3)	C7—C6—Fe1—C8	37.57 (13)
C5—C4—Fe1—C3	−118.7 (3)	C11—C6—Fe1—C8	156.5 (2)
C5—C4—Fe1—C8	165.07 (16)	C10—C6—Fe1—C2	165.56 (16)
C3—C4—Fe1—C8	−76.2 (2)	C7—C6—Fe1—C2	−76.01 (15)
C5—C4—Fe1—C2	−81.49 (19)	C11—C6—Fe1—C2	42.9 (2)
C3—C4—Fe1—C2	37.24 (17)	C10—C6—Fe1—C1	124.46 (15)
C5—C4—Fe1—C6	48.9 (3)	C7—C6—Fe1—C1	−117.11 (13)
C3—C4—Fe1—C6	167.7 (2)	C11—C6—Fe1—C1	1.8 (2)
C5—C4—Fe1—C1	−37.75 (17)	C10—C6—Fe1—C7	−118.42 (19)
C3—C4—Fe1—C1	80.98 (18)	C11—C6—Fe1—C7	118.9 (2)
C5—C4—Fe1—C7	−164.8 (3)	C2—C1—Fe1—C4	−81.93 (19)
C3—C4—Fe1—C7	−46.1 (4)	C5—C1—Fe1—C4	37.02 (19)
C10—C9—Fe1—C4	−115.05 (17)	C2—C1—Fe1—C9	−156.7 (3)
C8—C9—Fe1—C4	125.64 (17)	C5—C1—Fe1—C9	−37.8 (4)
C8—C9—Fe1—C10	−119.3 (2)	C2—C1—Fe1—C10	167.83 (15)
C10—C9—Fe1—C5	−75.16 (18)	C5—C1—Fe1—C10	−73.2 (2)
C8—C9—Fe1—C5	165.53 (15)	C2—C1—Fe1—C5	−118.9 (2)
C10—C9—Fe1—C3	−156.65 (17)	C2—C1—Fe1—C3	−38.36 (18)
C8—C9—Fe1—C3	84.04 (18)	C5—C1—Fe1—C3	80.6 (2)
C10—C9—Fe1—C8	119.3 (2)	C2—C1—Fe1—C8	47.0 (3)
C10—C9—Fe1—C2	170.9 (2)	C5—C1—Fe1—C8	166.0 (2)
C8—C9—Fe1—C2	51.6 (3)	C5—C1—Fe1—C2	118.9 (2)
C10—C9—Fe1—C6	37.72 (14)	C2—C1—Fe1—C6	125.67 (16)
C8—C9—Fe1—C6	−81.60 (15)	C5—C1—Fe1—C6	−115.39 (17)
C10—C9—Fe1—C1	−46.1 (4)	C2—C1—Fe1—C7	82.42 (17)
C8—C9—Fe1—C1	−165.4 (3)	C5—C1—Fe1—C7	−158.63 (16)
C10—C9—Fe1—C7	81.95 (15)	C8—C7—Fe1—C4	−38.8 (4)
C8—C9—Fe1—C7	−37.36 (13)	C6—C7—Fe1—C4	−158.1 (4)
C9—C10—Fe1—C4	81.92 (18)	C15—C7—Fe1—C4	82.6 (4)
C6—C10—Fe1—C4	−158.28 (14)	C8—C7—Fe1—C9	37.27 (15)
C6—C10—Fe1—C9	119.8 (2)	C6—C7—Fe1—C9	−82.02 (14)
C9—C10—Fe1—C5	123.64 (15)	C15—C7—Fe1—C9	158.7 (2)
C6—C10—Fe1—C5	−116.56 (15)	C8—C7—Fe1—C10	80.94 (15)
C9—C10—Fe1—C3	50.4 (3)	C6—C7—Fe1—C10	−38.35 (13)
C6—C10—Fe1—C3	170.2 (2)	C15—C7—Fe1—C10	−157.7 (2)
C9—C10—Fe1—C8	−37.62 (14)	C8—C7—Fe1—C5	165.5 (2)
C6—C10—Fe1—C8	82.17 (15)	C6—C7—Fe1—C5	46.2 (3)
C9—C10—Fe1—C2	−166.0 (3)	C15—C7—Fe1—C5	−73.1 (3)
C6—C10—Fe1—C2	−46.2 (4)	C8—C7—Fe1—C3	−74.46 (18)
C9—C10—Fe1—C6	−119.8 (2)	C6—C7—Fe1—C3	166.25 (14)
C9—C10—Fe1—C1	164.45 (15)	C15—C7—Fe1—C3	46.9 (2)
C6—C10—Fe1—C1	−75.75 (18)	C6—C7—Fe1—C8	−119.29 (18)
C9—C10—Fe1—C7	−81.48 (14)	C15—C7—Fe1—C8	121.4 (2)
C6—C10—Fe1—C7	38.32 (13)	C8—C7—Fe1—C2	−116.01 (15)
C1—C5—Fe1—C4	−119.5 (3)	C6—C7—Fe1—C2	124.70 (14)
C4—C5—Fe1—C9	−73.4 (2)	C15—C7—Fe1—C2	5.4 (2)
C1—C5—Fe1—C9	167.07 (16)	C8—C7—Fe1—C6	119.29 (18)
C4—C5—Fe1—C10	−114.5 (2)	C15—C7—Fe1—C6	−119.3 (2)
C1—C5—Fe1—C10	125.96 (17)	C8—C7—Fe1—C1	−158.82 (15)
C4—C5—Fe1—C3	37.86 (19)	C6—C7—Fe1—C1	81.89 (15)
C1—C5—Fe1—C3	−81.7 (2)	C15—C7—Fe1—C1	−37.4 (2)
C4—C5—Fe1—C8	−42.1 (4)	C17—C16—N1—C15	161.4 (3)
C1—C5—Fe1—C8	−161.6 (3)	C18—C16—N1—C15	−74.6 (3)
C4—C5—Fe1—C2	81.8 (2)	C7—C15—N1—C16	−166.5 (2)
C1—C5—Fe1—C2	−37.71 (17)	C6—C11—N2—C13	−67.2 (3)
C4—C5—Fe1—C6	−157.39 (18)	C12—C11—N2—C13	61.4 (3)
C1—C5—Fe1—C6	83.07 (19)	C6—C11—N2—C14	165.4 (2)
C4—C5—Fe1—C1	119.5 (3)	C12—C11—N2—C14	−65.9 (3)
C4—C5—Fe1—C7	169.3 (2)		

### Table 1 Table 1

Selected torsion angles (°)

**Table:**

C1—Cg1—Cg2—C6	7.60	C4—Cg1—Cg2—C9	7.51
C2—Cg1—Cg2—C7	7.06	C5—Cg1—Cg2—C10	7.16
C3—Cg1—Cg2—C8	7.38		

Cg1 is the centroid of the C1–C5 Cp ring and Cg2 is the centroid of the C6–C10 Cp ring.
